# Datasets of social capital and business performance in the Nigerian informal sector

**DOI:** 10.1016/j.dib.2021.106918

**Published:** 2021-02-27

**Authors:** Olamide Oluwabusola Akintimehin, Anthony Abiodun Eniola, Damilola Felix Eluyela, Rose Ogbechie

**Affiliations:** a Department of Business Ethics, Lagos Business School, Nigeria; b Department of Business Administration, Landmark University, Nigeria; c Department of Accounting and Finance, Landmark University, Nigeria; d Landmark University SDG 8 (Decent Work and Economic Growth Research Group), Nigeria

**Keywords:** Business, Business performance, Informal sector, Social capital, Nigeria

## Abstract

This research aimed to present data on the effect of social capital on business performance in the Nigerian informal economy. Primary data collection was carried out through a cross-sectional survey of 600 informal business owners within Ikeja Local Government Area (LGA), Lagos State, Nigeria. A simple sampling technique was further adopted in selecting the sample size of the study, and a close-ended questionnaire was adopted for the data collection process. Descriptive statistical analysis was performed using the Statistical Package for Social Sciences (SPSS) version 23. This data has the potential to be reused for full empirical research relating to social capital and business performance in emerging economies.

## Specifications Table

**Table:** 

Subject	Business, Management and Decision Sciences
Specific subject area	Entrepreneurship; Marketing
Type of data	Table Figure
How data were acquired	Cross-sectional survey; Self-administered questionnaire
Data format	Raw Analyzed
Parameters for data collection	The adoption of a cross-sectional survey method in collecting primary data from informal business owners in Lagos State, Nigeria, through the usage of self-administered close-ended questionnaires
Description of data collection	Data collection was done through a simple random sample of 600 informal business owners
Data source location	Ikeja Local Government Area, Lagos State Nigeria
Data accessibility	With the article https://data.mendeley.com/datasets/6wnmchnc6j/1
Related research article	Akintimehin, O. O., Eniola, A. A., Alabi, O. J., Eluyela, D. F., Okere, W., & Ozordi, E. (2019). Social capital and its effect on business performance in the Nigerian informal sector. Heliyon, 5(7). https://doi.org/10.1016/j.heliyon.2019.e02024

## Value of the Data

• This dataset is important to the research community in understanding the effect of internal and external social capital resources on the financial and non-financial performance of small and medium enterprises (SMEs) in the Nigerian informal sector. To the best of our knowledge, this is a complete dataset available for measuring the effect of social capital resources on the business performance of SMEs in the informal sector of an emerging economy.
• This dataset is valuable for researchers who want to conduct comparative studies related to social capital and its effect on business performance in other emerging or developed economies around the world, especially among informal entrepreneurs, or to make meta-analytic contributions in the future.
• This dataset is useful for researchers who intend to reuse or reanalyze it to investigate the possible relationships between social capital resources and business performance in the informal sector of an emerging economy [1]. For instance; to examine the comparative effect of internal or external social capital resources on the financial or non-financial performance (or both) of male and female-owned enterprises [2]; to examine the comparative effect of internal or external social capital resources on the business performance of family-owned and non-family-owned enterprises; or to examine the comparative effect of internal or external social capital resources on the financial or non-financial performance of sole proprietorship-based or partnership-based enterprises.
• The potentials of this dataset when analyzed through inferential statistical analysis, are valuable to enhancing entrepreneurs’ and policymakers’ awareness level regarding the importance of internal and external social capital resources in enhancing the financial and non-financial performance of SMEs in the informal sector of an emerging economy.
• This dataset is important to the research community (comprising researchers, entrepreneurs, policymakers and entrepreneurship students) because it has the potential to contribute to existing knowledge on the subject of social capital and SME performance in an emerging economy.

## 1 Data Description

This dataset provides insightful information based on survey data on internal and external social capital resources and their effect on the financial and non-financial performance of SMEs in the Nigerian informal sector. The survey involved 600 entrepreneurs within Ikeja Local Government Area (LGA), Lagos State, Nigeria. The data include six major variable groups, represented from Tables 1–6, thus: (A) Individual demographics: including age, gender, duration of firm existence, firm industry, firm ownership structure, firm ownership type, business engagement level and firm employee size; (B) 9 items measuring internal social capital, comprising family and friends. A five-point Likert scale was utilized in measuring these items (where 1= strongly disagree; 3= neutral, and 5= strongly agree); (C) 3 items measuring internal social capital, comprising business partners and employees. A five-point Likert scale was utilized in measuring these items (where 1= strongly disagree; 3= neutral; and 5= strongly agree); (D) 8 items measuring external social capital. A five-point Likert scale was utilized in measuring these items (where 1= strongly disagree; 3= neutral, and 5= strongly agree); (E) 4 items measuring financial performance. A five-point Likert scale was utilized in measuring these items (where 1= much worse; 3= about the same; and 5= much better); (F) 7 items measuring non-financial performance. A five-point Likert scale was utilized in measuring these items (where 1= much worse; 3= about the same; and 5= much better).

**Table 1:** Demographic features of the participants and their enterprises (*n* = 600).

Item	Frequency	Percentage (%)
**Gender**		
Male	262	43.7
Female	338	56.3
Total	600	100
**Age**		
Below 21years	29	4.8
21–29years	153	25.5
Above 30years	418	69.7
Total	600	100
**Duration of Firm Existence**		
1–2years	147	24.5
3–4years	178	29.7
5–6years	163	27.2
Above 6years	112	18.7
Total	600	100
**Firm Industry**		
Manufacturing	78	13
Wholesale and Retail Selling	80	13.3
Core Informal Services	318	53
Others	124	20.7
Total	600	100
**Firm Ownership Structure**		
Sole Proprietorship	443	73.8
Partnership	141	23.5
Others	16	2.7
Total	600	100
**Firm Ownership Type**		
Family-owned	124	20.7
Non family-owned	476	79.3
Total	600	100
**Business Engagement Level**		
Full-time entrepreneurs	467	77.8
Part-time entrepreneurs	133	22.2
Total	600	100
**Firm Employee Size**		
0–3 employees	303	50.5
4–7 employees	253	42.2
Above 7 employees	44	7.3
Total	600	100

Demographic features of the respondents are presented in Table 1, while the comprehensive assessment of responses on internal social capital (comprising family and friends), internal social capital (comprising business partners and employees), external social capital, financial performance and non-financial performance by the respondents are presented in Tables 2–6.

**Table 2:** Internal social capital (family and friends) (*n* = 9 items).

	Strongly								Strongly	
	Disagree		Disagree		Neutral		Agree		Agree	
Item	Freq(n)	%	Freq(n)	%	Freq(n)	%	Freq(n)	%	Freq(n)	%
family members offer financial support for the firm when needed	53	8.8	231	38.5	26	4.3	125	20.8	165	27.5
friends/colleagues offer soft loans for the firm when needed	42	7	245	40.8	59	9.8	144	24	110	18.3
family members offer strategic business advice	14	2.3	36	9	28	4.7	331	55.2	191	31.9
we get referrals through family members	12	2	32	5.3	27	4.5	332	55.3	197	32.8
we get referrals through friends/colleagues	9	1.5	27	4.5	26	4.3	314	52.3	224	37.3
friends/colleagues patronize our business as much as possible	12	2	23	3.8	28	4.7	326	54.3	211	35.2
family members patronize our business as much as possible	13	2.2	23	3.8	36	6	323	53.8	205	34.2
Family members promote our business activities as much as possible	21	3.5	43	7.2	53	8.8	350	58.3	133	22.2
friends/colleagues engage in mental collaborations with us concerning the business	20	3.3	154	25.7	172	28.7	161	26.8	93	15.5

**Table 3:** Internal social capital (business partners and employees) (*n* = 3 items).

	Strongly								Strongly	
	Disagree		Disagree		Neutral		Agree		Agree	
Item	Freq(n)	%	Freq(n)	%	Freq(n)	%	Freq(n)	%	Freq(n)	%
Business partners share the same ambition for the firm	10	1.7	50	8.3	264	44	173	28.8	103	17.2
Employees/apprentices trust the product/ service offerings of the business	14	2.3	39	6.5	105	17.5	293	48.8	149	24.8
The firm's vision, mission and values are understood and driven by all business associates involved	9	1.5	41	6.8	264	44	150	25	136	22.7

**Table 4:** External social capital (*n* = 8 items).

	Strongly								Strongly	
	Disagree		Disagree		Neutral		Agree		Agree	
Item	Freq(n)	%	Freq(n)	%	Freq(n)	%	Freq(n)	%	Freq(n)	%
We have a fantastic relationship with our customers	4	0.7	27	4.5	28	4.7	138	23	403	67.2
We have a fantastic relationship with our suppliers	2	0.3	33	5.5	34	5.7	161	26.8	370	61.7
We enjoy referrals through our existing customers	10	1.7	29	4.8	43	7.2	229	38.2	289	48.2
Our customers trust our product/service offerings	9	1.5	24	4	34	5.7	318	53	215	35.8
Customers offer us vital market information and strategic business advice	13	2.2	22	3.7	49	8.2	348	58	168	28
We enjoy special discounts from our suppliers	7	1.2	34	5.7	61	10.2	346	57.7	152	25.3
Our customers suggest to us how we can better satisfy them	8	1.3	21	3.5	35	5.8	275	45.8	261	43.5
We get easy access to market information from our suppliers	11	1.8	26	4.3	48	8	323	53.8	192	32

**Table 5:** Financial performance (*n* = 4 items).

	Much		Slightly		About		Slightly		Much	
	worse		worse		the same		better		better	
Item	Freq(n)	%	Freq(n)	%	Freq(n)	%	Freq(n)	%	Freq(n)	%
Our revenue earnings in comparison with that of competitors	45	7.5	26	4.3	195	32.5	191	31.8	142	23.9
Our market share in comparison with that of competitors	28	4.7	39	6.5	140	23.3	236	39.3	157	26.2
Our returns on investment in comparison with that of competitors	29	4.8	41	6.8	112	18.7	242	40.3	176	29.3
Our overall financial performance in comparison with that of competitors	23	3.8	38	6.3	101	16.8	239	39.8	199	33.2

**Table 6:** Non-financial performance (*n* = 7 items).

	Much		Slightly		About		Slightly		Much	
	worse		worse		the same		better		better	
Item	Freq(n)	%	Freq(n)	%	Freq(n)	%	Freq(n)	%	Freq(n)	%
Our product/service quality in comparison with that of competitors	28	4.7	40	6.7	146	24.3	195	32.5	191	31.8
Our customer satisfaction rate in comparison with that of competitors	22	3.7	40	6.7	113	18.8	238	39.7	187	31.2
Our customer preference rate in comparison with that of competitors	26	4.3	33	5.5	120	20	228	38	193	32.2
Our customer loyalty rate in comparison with that of competitors	18	3	39	6.5	143	23.8	220	36.7	180	30
Our product/service innovation rate in comparison with that of competitors	26	4.3	34	5.7	102	17	225	37.5	213	35.5
Our market size in comparison with that of competitors	22	3.7	38	6.3	99	16.5	208	34.7	233	38.8
Our competitive position in comparison with that of competitors	38	6.3	36	6	121	20.2	162	27	243	40.5

This dataset has already been used to publish a full research article [1], while the raw dataset is provided as a supplementary file and can also be found in the Mendeley data repository [3].

## 2 Experimental Design, Materials and Methods

The researchers adopted a cross-sectional survey design in obtaining primary data from 650 informal business owners in the Nigerian informal sector. Data was gathered using a close-ended research questionnaire and distributed across these informal enterprises within the Ikeja LGA, Lagos State, Nigeria, within a six-week time frame, using the sample random sampling technique. Out of these 650 questionnaires administered, 50 were discarded due to non-completion or incorrect completion by the respondents. The final 600 responses were analyzed through descriptive statistical analysis, using the Statistical Package for Social Sciences (SPSS) version 23 [1].

The questionnaire used in data collection was divided into two sections: Section A and Section B. Section A was used to obtain demographic information from the study respondents. In contrast, Section B was used to assess the subjective contributions of internal and external social capital resources to the financial and non-financial performance of SMEs in the Nigerian informal sector.

## Ethics Statement

Ethical approval was obtained from the Landmark University Centre for Research, Innovation and DiscoverEthical Committee. Also, informed consent was obtained from the research participants and their participation was totally consensual, anonymous and voluntary.

## Data Availability

Datasets of social capital and business performance in the Nigerian informal sector (Original data) (Mendeley Data).

## Declaration of Competing Interest

The authors declare that they have no known competing financial interests or personal associations which have, or could be perceived to have, influenced the work described in this article.

## Appendix Supplementary materials

Image, application 1

Image, application 2

Image, application 3
