# Supplementary material for: Datasets of social capital and business performance in the Nigerian informal sector
Source: Data Brief. 2021 Feb 27;35:106918. doi: 10.1016/j.dib.2021.106918 (PMC7966821; doi:10.1016/j.dib.2021.106918)
Supplement: Supplementary file 3 [file mmc3.docx]

**RESEARCH QUESTIONNAIRE**

**PART ONE**

**Instruction: Please tick [**√**] and fill in as appropriate.**

1. Gender: (a) Male [ ] (b) Female [ ]
2. Age: (a) Below 21 years [ ] (b) 21 – 29 years [ ] (c) above 30 years [ ]
3. Duration of firm existence: (a) 1-2years [ ] (b) 3-4years [ ] (c) 5-6years [ ] (d) above 6years [ ]
4. Firm ownership structure: (a) Sole-proprietorship [ ] **(**b**)** partnership [ ] **(**c**)** other [ ]
5. Firm ownership type: (a) family owned [ ] (b) non-family owned [ ]
6. Industry of operation: (a) manufacturing [ ] (b) wholesale and retail selling [ ] (c) core informal services [ ] (d) others
7. Form of business engagement: (a) full-time engagement [ ] (b) part-time engagement (as a side hustle) [ ]
8. firm size: (a) 0-3 employees [ ] (b) 4-7 employees [ ] (c) above 7 employees [ ]

**PART TWO**

**Instruction: Please tick [**√**] as it tallies with your answer.**

Where:

**SA = Strongly Agree; A = Agree; N = Neutral; D = Disagree; SD = Strongly Disagree**

| **Internal Social Capital (Family and Friends)** | **SA** | **A** | **N** | **D** | **SD** |
| --- | --- | --- | --- | --- | --- |
| family members offers financial support for the firm when needed |  |  |  |  |  |
| friends/colleagues offer soft loans for the firm when needed |  |  |  |  |  |
| family members offers strategic business advice |  |  |  |  |  |
| we get referrals through family members |  |  |  |  |  |
| we get referrals through friends/colleagues |  |  |  |  |  |
| friends/colleagues patronize our business as much as possible |  |  |  |  |  |
| family members patronize our business as much as possible |  |  |  |  |  |
| Family members promote our business activities as much as possible |  |  |  |  |  |
| friends/colleagues engage in mental collaborations with us concerning the business |  |  |  |  |  |
| **Internal Social Capital (Business Partners and Employees)** |  |  |  |  |  |
| Business partners share the similar ambition for the firm |  |  |  |  |  |
| Employees/apprentices trust the product/service offerings of the business |  |  |  |  |  |
| The firm’s vision, mission and values are understood and driven by all business associates involved |  |  |  |  |  |
| **External Social Capital** | **SA** | **A** | **N** | **D** | **SD** |
| We have a fantastic relationship with our customers |  |  |  |  |  |
| We have a fantastic relationship with our suppliers |  |  |  |  |  |
| We enjoy referrals through our existing customers |  |  |  |  |  |
| Our customers trust our product/service offerings |  |  |  |  |  |
| Customers offer us vital market information and strategic business advice |  |  |  |  |  |
| We enjoy special discounts from our suppliers |  |  |  |  |  |
| Our customers suggest to us how we can better satisfy them |  |  |  |  |  |
| We get easy access to market information from our suppliers |  |  |  |  |  |
| **MW= much worse; SW= slightly worse; AS= about the same; SB=slightly better; MB=much better** | | | | | |
| **Financial Performance** | **MW** | **SW** | **AS** | **SB** | **MB** |
| Our revenue earnings in comparison with that of competitors |  |  |  |  |  |
| Our market share in comparison with that of competitors |  |  |  |  |  |
| Our returns on investment in comparison with that of competitors |  |  |  |  |  |
| Our overall financial performance in comparison with that of competitors |  |  |  |  |  |
| **Non-financial Performance** | **MW** | **SW** | **AS** | **SB** | **MB** |
| Our product/service quality in comparison with that of competitors |  |  |  |  |  |
| Our customer satisfaction rate in comparison with that of competitors |  |  |  |  |  |
| Our customer preference rate in comparison with that of competitors |  |  |  |  |  |
| Our customer loyalty rate in comparison with that of competitors |  |  |  |  |  |
| Our product/service innovation rate in comparison with that of competitors |  |  |  |  |  |
| Our market size in comparison with that of competitors |  |  |  |  |  |
| Our competitive position in comparison with that of competitors |  |  |  |  |  |
